# Notes and Notices

**Published:** 1891-10

**Authors:** 


### NOTES AND NOTICES.

Dr. Frank Kraft has withdrawn from the Cleveland Homœopathic Hospital College.

Dr. Skinner has returned to his rooms in London *for one month* from the 7th of September before he goes to Holland for another month. All consultations must be by appointment.

Dr. C. Eurich removed from 119 East Eighty-sixth Street to 124 East Eighty-fifth Street, between Lexington and Park Avenues, New York City.

Dr. Eyermann has removed his residence and office to 1722 South Jefferson Avenue, St. Louis, Mo.; he also has a branch office at 3921 South Broadway.

Dr. G. J. Waggoner has removed from Minonk, Ill., to 520 Minnesota Avenue, Kansas City, Kan.

Dr. L. B. Wells has removed from Park Avenue to 31 Summit Place, Utica, N. Y.

Messrs. Reed & Carnrick have rebuilt their laboratory, and are better prepared than before their big fire to furnish the excellent specialties which bear their name. In this connection we invite special attention to their new advertisement. They are known everywhere, and their name is the synonym for fair dealing and scientific pharmacy.—*Practice.*

Printers' Ink.—If the *Register* could induce every merchant and manufacturer in the hillside city to become patrons of *Printers' Ink* for three months, we feel certain that they would be induced to expend more cash in the use of printers' ink, as used by the local printers, and receive returns commensurate with which to continue the same.—*Register*, Newburgh, N. Y., April 10th.

The Fabiola is the name of a new Sanitarium and private infirmary opened at 486 North Flores Street, San Antonio., Tex., by Dr. C. E. Fisher, editor of *The Southern Journal of Homœopathy.* Write to him for prospectus.

